# Influence of Fructooligosaccharide on Pharmacokinetics of Isoflavones in Postmenopausal Women

**DOI:** 10.1155/2012/783802

**Published:** 2012-04-26

**Authors:** Supanimit Teekachunhatean, Sujitra Techatoei, Noppamas Rojanasthein, Maleeya Manorot, Chaichan Sangdee

**Affiliations:** ^1^Department of Pharmacology, Faculty of Medicine, Chiang Mai University, Chiang Mai 50200, Thailand; ^2^Center of Thai Traditional and Complementary Medicine, Faculty of Medicine, Chiang Mai University, Chiang Mai 50200, Thailand

## Abstract

The objective of the present paper was to determine the influence of fructooligosaccharide (FOS) on the pharmacokinetics of isoflavones in healthy postmenopausal women. The study was a fixed-sequence, two-phase, crossover study. Twelve subjects received a single oral dose of 300 mL of a soy beverage. Blood samples were collected before the dose and at 0.5, 1, 2, 4, 6, 8, 10, 12, 24, and 32 h after the administration of the soy beverage. After a washout period of at least 1 week, subjects were assigned to receive oral doses of FOS, 5 g each time, twice a day (after breakfast and dinner) for 14 days, followed by a single oral dose of the same soy beverage on the next day. Blood samples were then collected at the same time points mentioned previously. Plasma isoflavone concentrations were determined by HPLC. Continuous oral administrations of FOS followed by a single oral administration of soy beverage caused significant increases in *C*_max_, AUC_0–32_, and AUC_0–∞_ of genistein and AUC_0–32_ of daidzein, comparing to those obtained following a single oral dose of soy beverage alone. Other pharmacokinetic parameters (*T*_max_ and *t*_1/2_ of both aglycones and AUC_0–∞_ of daidzein) between both regimens were not significantly different.

## 1. Introduction

Decreased estrogen level demonstrated in postmenopausal women commonly relates to a variety of disorders, for example, hot flushes, osteoporosis, depression, increased body weight, and so forth. As a therapeutic goal, hormone replacement therapy (HRT) has been used for decades as the “gold standard” to treat estrogen deficiency symptoms [1]. Estrogen therapy has been demonstrated to benefit postmenopausal women mostly through the relief of vasomotor symptoms as well as for the prevention and treatment of osteoporosis [2]. However, estrogen therapy has been shown to increase the risk of breast cancer, uterine and endometrial cancer, as well as menstrual bleeding [3]. Although the incidence of endometrial cancer can be avoided by using estrogen plus progestin [4], this combination increases the risks of stroke, pulmonary embolism, and invasive breast cancer [5]. Therefore, both postmenopausal women and physicians remain concerned about the adverse effects of estrogen and are looking for natural products that possess beneficial effects similar to estrogen but have more favorable safety profiles. Indeed, phytoestrogens offer attractive options because they have been demonstrated to decrease the risk of endometrial and breast cancer, avoid the need for progestin, have fewer adverse reactions, and still provide health benefits [6].

Isoflavones represent the most common group of phytoestrogens, and soybeans are the most common source of isoflavones in human food. Isoflavones are structurally similar to estrogen but possess weaker hormonal effects. They bind weakly to the estrogen receptor alpha (ER*α*) of the reproductive organs such as the uterus, ovaries, and breast, but bind more strongly to the estrogen receptor beta (ER*β*) found in the brain, arteries and bone [7], and have been associated with beneficial effects in humans, such as the relief of hot flushes, and the prevention of osteoporosis, cardiovascular diseases, and cancers [8].

 The unconjugated aglycones (daidzein, genistein, and glycitein) are contained predominantly in fermented soy-based products (such as tempeh and miso) due to microorganism-induced hydrolysis of their respective glycoside conjugates into aglycones, whereas most nonfermented soy-based products (such as tofu, soymilk, soy flour, etc.) largely contain mixtures of their malonyl-, acetyl-, and nonconjugated *β*-glycosides [9]. All glycosides are biologically inactive and poorly absorbed from the intestinal tract because of their hydrophilicity and larger molecular size [10]. Hydrolysis of the glycosidic bond using *β*-glucosidases is therefore necessary to release the biologically active aglycones which are rapidly absorbed across the intestinal wall [7, 10, 11]. The *β*-glucosidases involved in this step are found in the small intestinal brush border (lactase phloridzin hydrolase ([LPH])) [12] and within enterocytes (cytosolic *β*-glucosidases) [13]. Nonetheless, isoflavone glycosides that are not absorbed in the small intestine will pass through to the colon, where bacterial *β*-glycosidases can hydrolyze them and additional absorption can be exerted [10, 14–16]. Several groups of colonic bacteria including *Lactobacillus* spp., *Bacteroides* spp., and *Bifidobacterium* spp. possess *β*-glucosidase activity and have been demonstrated to be very effective deglycosylators [14].

Prebiotics such as fructooligosaccharides (FOSs) are poorly digested in the human small intestine but are fermented in the colon by the resident microflora [17]. FOS is also found to be fermented *in vitro* only by a specific range of microflora that include most species of bifidobacteria [18, 19]. The oral administration of FOS for 7 days has been reported to significantly increase fecal bifidobacteria counts in healthy volunteers [20]. As aforementioned, this increased number of colonic bifidobacteria theoretically leads to an enhancement of bacterial *β*-glucosidase activity in the colon. It is possible that supplementation with FOS could increase the isoflavone absorption through enhancement of bacterial *β*-glucosidase activity [15]. Uehara et al. have shown that plasma and urine levels of genistein and daidzein are higher in the rat fed with FOS than the control rat [21]. In addition, an additive effect of dietary isoflavones and FOS has been demonstrated on the bone mineral density in ovariectomized mice [22]. However, the study of FOS on plasma isoflavone concentrations in human is still lacking. Therefore, the purpose of this study was to determine the influence of FOS on the pharmacokinetics of isoflavones in healthy, Thai postmenopausal women.

## 2. Materials and Methods

### 2.1. Study Design

 The study was a fixed-sequence, two-phase study with a washout period of at least one week. The study was approved by the Human Research Ethics Committee of the Faculty of Medicine, Chiang Mai University, and complied with the Helsinki Declaration.

### 2.2. Subjects

 A total of 12 Thai postmenopausal women, age >45 y, postmenopausal status >1 y (since the last spontaneous menstrual bleeding), and serum follicle-stimulating hormone concentration >30 IU/L, were enrolled in this study. The body mass index (BMI) of subjects was within 18–25 kg/m^2^. All had to be in good health based on medical history, physical examination, and routine blood tests including complete blood count, blood urea nitrogen, creatinine, and liver function tests. Exclusion criteria were history of malignancy, chronic renal, liver, cardiovascular, pulmonary or breast disease, a history of substance abuse or addiction, recent cigarette smoking, regular consumption of >2 alcoholic drinks/d, regular use of over-the-counter or prescription medication (>1 dose/wk), use of antibiotics and laxatives within the previous 4 weeks, and intake of nutritional supplements (containing vitamins, minerals, fibers, FOS, isoflavones, other prebiotics or probiotics) within the previous 2 weeks.

### 2.3. Soy Preparation and FOS

 The soy preparation used in this study was a commercial instant soy beverage D in 1 (manufactured by T.C. Pharmaceutical, Co., Ltd., Thailand), prepared by mixing 1 sachet (36 g) of soy powder with 300 mL of hot water. The mean isoflavone contents of daidzin and genistin were 11.24 ± 2.84 and 23.82 ± 1.03 mg/serving, respectively. The amounts of daidzein and genistein were negligible. FOS used in this study was Meioligo granule 2.5 g/sachets, manufactured by Meiji Seika Kaisha, Ltd., Tokyo, Japan.

### 2.4. Dosage and Drug Administration

Subjects were admitted to the Clinical Pharmacology Unit of the Faculty of Medicine, Chiang Mai University, at 6:30 AM after an overnight fast of at least 8 h. Each received a single oral dose of 300 mL soy beverage (D_0_) (single ISO phase). The subjects were instructed to remain upright and fast for 2 h after administration of the soy beverage. Water and lunch were served at 2 h and 6 h after dosing, respectively. Blood samples were collected at specific time points (see the following). Following blood sample collection at 12 h after the soy dose, the subjects were discharged from the Clinical Pharmacology Unit and were asked to come back again on the next day for blood sample collections at 24 h and 32 h after the initial dose. After a washout period of at least 1 week, subjects received oral doses of FOS, 5 g each time, twice a day (after breakfast and dinner) for 14 days (D_−14_–D_−1_), followed by a single oral dose of 300 mL soy beverage on the next day (D_0_) (continuous FOS/single ISO phase). Administrations of soy beverage and blood sample collections were performed in the same manner as in the former phase. An identical food and beverage containing no isoflavones were served during both phases. Subjects were required to refrain from drinking some beverages (e.g., soy milk, alcohol and caffeine containing beverages, etc.).

### 2.5. Blood Samples Collection

 Venous blood samples (7 mL each) for determination of plasma isoflavones were collected before the dose, and then at 0.5, 1, 2, 4, 6, 8, 10, 12, 24, and 32 h after administration of the soy beverage. Samples were obtained from the forearm by venipuncture through an indwelling intravenous catheter and collected in a heparinized vacutainer. The blood collecting tubes were centrifuged at 1,200 rpm for 15 min and the plasma was separated and frozen at −80°C for later analysis.

### 2.6. Determination of Plasma Concentrations of Isoflavones

#### 2.6.1. Sample Preparation

The sample preparation and determination of isoflavone concentrations in plasma were modified from the method described by Teekachunhatean et al. [23]. An aliquot of 250 *μ*L of plasma was transferred to a 1.5 mL plastic vial and treated with 0.15 mL of a mixture of *β*-glucuronidase/sulfatase from *Helix pomatia* (Sigma G-0876) to hydrolyze glucuronide and sulfate conjugates of genistein and daidzein. The enzyme mixture was made up freshly and contained 0.1 g ascorbic acid in 10 mL of 0.1 M sodium acetate buffer, 0.01 g ethylenediaminetetraacetic acid (EDTA), and 250 *μ*L of *Helix pomatia*. The tubes containing the enzyme mixture were capped and heated overnight in a water bath (15–18 h, 37°C) and then were allowed to cool to room temperature.

**Figure 1:**
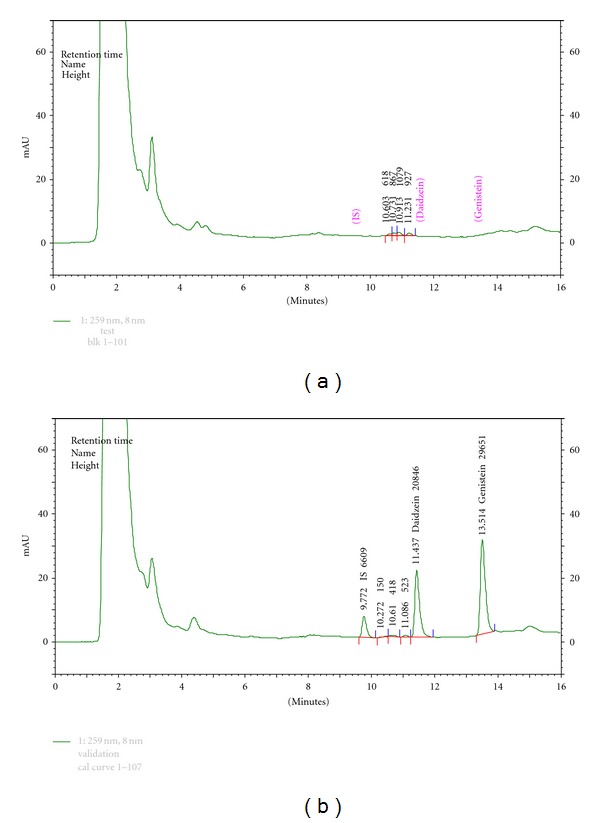
(a) Chromatogram of isoflavone-free plasma. (b) Chromatogram of plasma sample containing 2,400 ng/mL of daidzein (*k* = 11.437 min) and genistein (*k* = 13.514 min) as well as 10,000 ng/mL of IS (*k* = 9.772 min).

 After enzymatic hydrolysis, plasma samples were spiked with 10 *μ*L of internal standard (IS, 50,000 ng/mL fluorescein in 80% methanol) and then deproteinated by mixing the plasma sample with 1,000 *μ*L of 1% acetic acid in acetonitrile, vortex mixing for 30 sec, and centrifuged at 14,000 rpm for 10 min, respectively. An aliquot of the supernatant was removed and evaporated to vacuum dried for 3 h at 60°C. The residue was dissolved in 50 *μ*L of mobile phase B, and 5 *μ*L of the sample was injected into the HPLC system. Chromatogram of isoflavone-free plasma is shown in Figure 1(a), whereas chromatogram of plasma containing 2,400 ng/mL of daidzein and genistein as well as 10,000 ng/mL of IS is presented in Figure 1(b).

#### 2.6.2. High-Performance Liquid Chromatography (HPLC) Conditions

The assay of isoflavones was modified from the HPLC method and conditions previously described by Teekachunhatean et al. [23]. The samples were eluted on a C18 column (Inertsil, 150 mm × 4.6 I.D., 5 *μ*m, GL Science, Tokyo, Japan) with a C18 guard column (Inertsil ODS-3, 10 mm × 4.0 I.D., 5 *μ*m, GL Science, Tokyo, Japan). The chromatography condition consisted of two mobile phases. Mobile phase A was 55 mM ammonium acetate in deionized water/acetonitrile/methanol (250 : 45 : 45, v/v/v). Mobile phase B was 55 mM ammonium acetate in deionized water/acetonitrile/methanol (250 : 255 : 220, v/v/v). Both mobile phases contained 29 *μ*L of perchloric acid and 250 *μ*L of 1.44 mM sodium dodecyl sulfate. Separation was performed at 25°C. A gradient elution of 90% A with 10% B for 3.5 min, 50% A with 50% B at 3.5–6.5 min, 30% A with 70% B at 6.5–9.2, and 5% A with 95% B at 9.2–12.8 min was scheduled. The flow rate of mobile was maintained at 1 mL/min, and the analyses were detected by UV absorption at 259 nm. The isoflavone contents of samples were determined by using a calibration curve of peak height ratios of isoflavones and IS versus respective isoflavone concentrations (37.5, 75, 150, 300, 600, 1,200, and 2,400 ng/mL) with the use of linear regression. The linear regression analysis of peak height ratios of isoflavones versus isoflavone concentrations consistently yielded coefficients of determinant (*r* ^2^) of 0.997 or better.

For intraday validation, 5 samples from each of 3 quality control (QC) samples (112.5, 1,100, 2,200 ng/mL) were evaluated with a single calibration curve. For interday validation, 5 sets of the 3 different concentrations of QC samples (112.5, 1,100, 2,200 ng/mL) were studied on 5 independent days with concurrent 5 standard calibration curves. The precision was reported as the percentage of coefficient of variation (% CV) which was calculated as follows:

(1) $$\text{\%}\;\text{CV}=\frac{\text{SD}}{\overset{̅}{X}}\times 100,$$

where SD was standard deviation and $\overset{̅}{X}$ was mean value of isoflavone concentration in plasma.

 The deviation was expressed as the percentage of inaccuracy calculated by the following equation:

(2) $$\begin{array}{cc} & \text{\%}\;\text{Deviation}\\ & \;\;=\frac{\left(\text{Measured}\;\text{concentration}-\text{Spiked}\;\text{concentration}\right)}{\text{Spiked}\;\text{concentration}}\\ & \;\;\times 100. \end{array}$$

For the determination of daidzein concentrations in plasma, the % CV of intraday precision for the 3 QC samples was 2.65%, 1.36%, and 1.29%, respectively, whereas that of interday precision was 6.04%, 1.10%, and 3.05%, respectively. The % deviation of intraday assay for the 3 QC samples was −5.49%, −1.32%, and −1.07%, respectively, whereas that of interday precision was −2.40%, 0.61%, and 3.23%, respectively.

For the determination of genistein concentrations in plasma, % CV of intraday precision for the 3 QC samples was 4.48%, 1.71%, and 1.30%, respectively, whereas % CV of interday precision was 2.57%, 1.83%, and 3.34%, respectively. The % deviation of intraday assay for the 3 QC samples was 4.48%, 1.71%, and 1.30%, respectively, whereas those of interday precision was 2.52%, −0.40%, and 2.46%, respectively.

The mean recovery of daidzein and genistein from the determination procedure was 94.69 ± 3.10% and 92.54 ± 2.97%, respectively.

### 2.7. Data Analysis and Statistical Methods

#### 2.7.1. Pharmacokinetic Parameters

Maximal plasma concentration (*C *_max_, ng/mL) and time to reach peak concentration (*T*_max_, h) were obtained directly by visual inspection of each subject's plasma concentration-time profile. The area under the plasma concentration-time curve from time 0–32 h and 0–∞ h (AUC_0–32_ and AUC_0–∞_, ng·h/mL) as well as half-life (*t*_1/2_, h) was determined by noncompartmental analysis. The slope of the terminal log-linear portion of the concentration-time profile was determined by least-squares regression analysis and was used as the elimination rate constant (*k*_*e*_). The elimination half-life was calculated as 0.693/*k*_*e*_. The AUC from time zero to the last quantifiable point (AUC_0–32_) was calculated using the trapezoidal rule. Extrapolated AUC from time *t* to infinity (AUC_t–∞_) was determined as Ct/*k*_*e*_. Total AUC_0–∞_ was the sum of AUC_0–32_ + AUC_32–∞_.The calculation was performed by using the TopFit software version 2.0 for PC.

#### 2.7.2. Statistical Analysis

The pharmacokinetic parameters were presented as mean ± SD. The differences in the mean values of pharmacokinetic parameters obtained from both phases were compared by using the Wilcoxon's signed-rank test. A *P* value <0.05 was considered significant.

## 3. Results

 The demographic characteristics of 12 female subjects enrolled in the study are shown in Table 1. All subjects completed the study.

**Figure 2:**
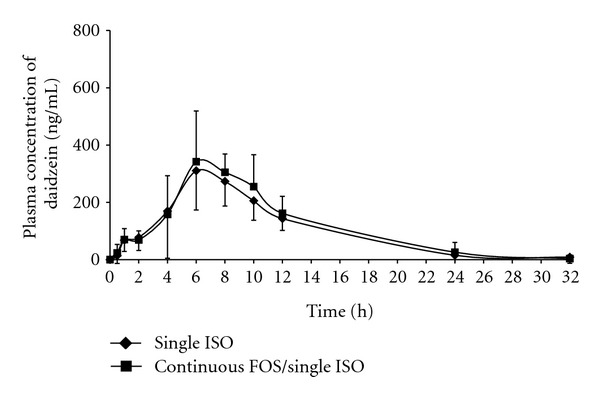
Mean plasma daidzein concentration-time curves from 12 subjects receiving a single oral administration of soy beverage (single ISO phase) and continuous oral administrations of FOS followed by a single oral administration of soy beverage (continuous FOS/single ISO phase).

**Figure 3:**
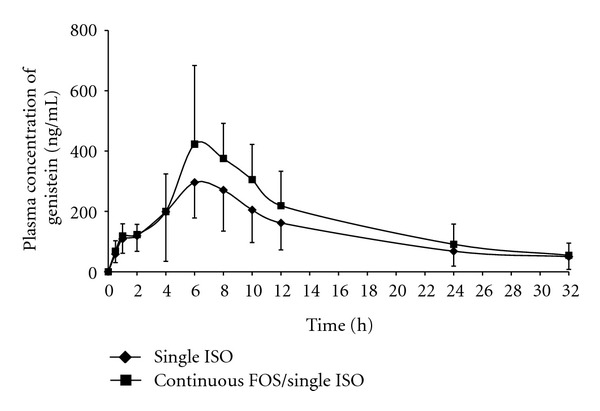
Mean plasma genistein concentration-time curves from 12 subjects receiving a single oral administration of soy beverage (single ISO phase) and continuous oral administrations of FOS followed by a single oral administration of soy beverage (continuous FOS/single ISO phase).

The mean plasma concentration-time curves of daidzein and genistein from 12 subjects receiving single ISO versus continuous FOS/single ISO are shown in Figures 2 and 3, respectively. The plasma concentration-time profiles of daidzein and genistein in both phases showed wide intraindividual and interindividual variation but were typically biphasic in every individual regardless of the regimens taken (data not shown). The first and second peak concentrations of daidzein and genistein obtained from both phases were approximately reached at 2–4 h and 6–8 h, respectively. The second peak concentrations of both isoflavones were always higher than the first peak.

 The pharmacokinetic parameters of daidzein and genistein (*C*_max_, AUC_0–32_, AUC_0–∞_, *T*_max_, *t*_1/2_) after oral administration of soy preparation in both phases were determined and are shown in Table 2. Of all pharmacokinetic parameters, it was shown that C_max_, AUC_0–32_, and AUC_0–∞_ of genistein as well as AUC_0–32_ of daidzein obtained from continuous FOS/single ISO phase were significantly higher than those of single ISO phase. For genistein, the mean *C*_max_ and AUC obtained from continuous FOS/single ISO phase were about 40% and 25%–30% and significantly higher comparing to those of “single ISO” phase. However, the mean AUC_0–32_ of daidzein obtained from “continuous FOS/single ISO” phase was only slightly (~15%) but statistically higher than that of “single ISO” phase. It is worth noting that significant increases in AUC_0–32_ of both aglycones in “continuous FOS/single ISO” phase were consistent with the higher second peaks of the plasma concentration-time curves, whereas the first peaks obtained from both phases were exactly the same.

 Data from “continuous FOS/single ISO” phase revealed that 4 out of 12 postmenopausal subjects demonstrated only minimal increases in AUC_0–32_ (1%–10% increment) of daidzein, but 7 out of 12 demonstrated remarkable increases in AUC_0–32_ (>10% increment). Similarly, 3 out of 12 demonstrated only minimal increases in AUC_0–32_ of genistein, and 8 out of 12 demonstrated remarkable increases in AUC_0–32_. Additionally, one subject demonstrated a decrease in AUC_0–32_ of both daidzein (−33%) and genistein (−16%).

## 4. Discussion

This is the first study to investigate the influence of FOS on pharmacokinetics of isoflavones in healthy postmenopausal women. Twelve subjects in this study were enrolled in accordance to the number of subjects in the previous study demonstrating that supplementation with prebiotic inulin significantly increases the extent of isoflavone absorption [24].

Despite the mixtures of malonyl-, acetyl-, and **β**-glycoside conjugates of daidzein and genistein are the major isoflavones found in most nonfermented soy-based products [16], both malonyl- and acetyl-conjugates can readily be hydrolyzed to their respective more heat-stable *β*-glycoside conjugates during exposure to processing temperature [9]. Hence this is the reason why daidzein and genistein were demonstrated to be the major forms of isoflavones in the instant soy beverage used in this study. The soy preparation containing the predominant isoflavone glycosides (daidzin and genistin) rather than aglycone forms was chosen because it has been hypothesized that FOS increases isoflavone bioavailability via stimulation of gut microflora ability to hydrolyze isoflavone glycosides to their respective more readily absorbable aglycones. Additionally, soy beverage was considered the formulation of choice rather than other commercially available soy extract capsules because the *β*-glycoside conjugates dissolved in soy beverage are ready to be hydrolyzed and further absorbed without the necessity of determining their disintegration and dissolution profiles, which are important confounding factors during absorption.

Although daidzin and genistin in the ingested soy beverage need to be hydrolyzed by the intestinal or gut microflora *β*-glucosidases to release free aglycones prior to absorption, the absorbed aglycones are further predominantly metabolized to their *β*-glucuronide conjugates, and to lesser amounts also as sulfate conjugates, in the intestine and/or liver [25]. Therefore, the glucuronide and sulfate conjugates are the major metabolites of isoflavones found in systemic circulation. Treating plasma samples with a mixture of *β*-glucuronidase/sulfatase before determination of plasma isoflavone concentrations in this study resulted in enzymatic hydrolysis of glucuronide and sulfate conjugates to aglycones (daidzein and genistein). Thus, plasma concentrations of the respective aglycones were determined rather than their glucuronide and sulfate conjugates [23].

The mean *T*_max_ of daidzein (6.67 ± 1.78 h) and genistein (6.00 ± 1.91 h) obtained from single ISO phase of this study was quite shorter than that of 8–11 h after ingestion of isoflavone conjugates reported in other studies [26, 27]. This discrepancy might be as a result of such factors as ethnic background, soy preparations used, intestinal microflora, and dietary habits. However, the *T*_max_ reported here was comparable to that of our previous studies in Thai postmenopausal women [23, 28]. Additionally, the mean *t*_1/2_ of daidzein and genistein was 4.65 ± 2.46 h and 9.61 ± 4.45 h, respectively, which are in agreement with those of 3–9 h for daidzein and 8–11 h for genistein after the intake of soy foods or pure isoflavone glycosides [29, 30].

In this study, a typical biphasic pattern appeared in both phases for all individuals, as well as in mean plasma concentrations-time curves of postmenopausal subjects. This finding is consistent from those reported elsewhere [26, 29, 31]. Plasma concentrations of isoflavones could be detected as early as 30 min, and the first peak was attained at approximately 2–4 h after soy intake. It has been reported that this early detection of plasma levels and the presentation of the first peak correspond to the hydrolysis of *β*-glycoside conjugates and initial absorption of aglycones readily occurring in the duodenum and proximal jejunum [26, 29, 32, 33]. Additionally, the second peak attained at 6–8 h corresponds to an ability of gut microflora to cleave *β*-glycosides to their respective aglycones before the latter can be absorbed mainly in the large intestine [10, 14–16]. The large intestine is known to be the location where resident microflora are present in the largest numbers and consequently play the most crucial role for the uptake of isoflavones [33]. The significant delay of the second peak after the radical reduction of the gut flora, achieved by mechanical bowel preparation in combination with oral antibiotic treatment, emphasizes further evidence that this peak corresponds to colonic bacterial *β*-glucosidase activity [33].

 In the “continuous FOS/single ISO” phase of this study, the significant increases in *C*_max_ (of genistein) and AUC (of both daidzein and genistein), compared to those of “single ISO” phase, correlated to the higher second peaks of the plasma concentration-time curves. These findings suggest that pretreatment of FOS for 14 days might efficiently lead to stimulation of microflora growth especially in the colon, resulting in enhancement of microflora *β*-glucosidases activity, and hence increased oral absorption of both aglycones. These are consistent with the previous study, which demonstrated that oral administration of FOS significantly increases the absorption of both aglycones in rats, especially during 6–48 h after isoflavone intake [21]. Similarly, study in healthy postmenopausal women has shown that plasma concentrations of both aglycones are significantly higher after the oral consumption of isoflavones plus prebiotic inulin for 21 days, compared to those of without inulin [24].

 The present investigation revealed that the majority of study subjects demonstrated remarkable increases in AUC (>10% increment) of both aglycones, whereas the minority (approximately 40% of participants) showed minimal increases or even decrease in these parameters. This discrepancy reflects the different potential of FOS to stimulate gut microflora growth, which might be due mainly to variations in basal amount and type of resident microflora responsible for isoflavone conversion, as well as their ability to metabolize glycoside conjugates in the intestinal milieu of different individuals [34]. Although FOS seems likely to stimulate a limited range of microorganisms, especially bifidobacteria [18], it is speculated that the growth of other bacterial species in some individuals might be simultaneously stimulated, leading to an increase in the biotransformation of isoflavones to metabolites other than their respective aglycones; for example, daidzein can be further metabolized to equol and *O*-desmethylangolensin, and genistein to p-ethyl phenol [16]. Indeed, it appears that more than one bacterial species could be involved in the metabolic conversions of these aglycones [35]. On the other hand, it is still uncertain whether an increased amount of bifidobacteria under some specific circumstances could further metabolize daidzein and genistein to other constituents resulting in lower oral bioavailability of these aglycones in some individuals. Therefore, the correlation between changes in isoflavone bioavailability versus the fecal bacterial counts (including bifidobacteria and others) and/or *β*-glucosidase activity in the fecal contents after FOS supplement should be further investigated, to clarify the unanswered question of how FOS affects the bacterial growth especially in subjects whose isoflavone bioavailability cannot be increased or even decreased.

 Interestingly, present and previous studies have unanimously demonstrated that FOS or inulin remarkably enhances the absorption of genistein, rather than daidzein both in rats [21], and in human subjects [24]. Since the combination of dietary FOS and isoflavones has been shown to correlate with increased plasma equol levels in rats [36, 37], and 30% of humans can metabolize daidzein to equol that is detectable in systemic circulation [15], it is hypothesized that enhancement of the conversion of daidzein to equol (and/or *O*-desmethylangolensin) from increased bacterial growth following FOS supplement could explain why it is less likely to increase the bioavailability of daidzein in comparison to genistein. Nonetheless, the role of bifidobacteria involving in the reductive pathway of daidzein toward equol is still controversial. Although the formation of equol in soymilk fermented with some strains of bifidobacteria has been reported [38], 22 strains of bifidobacteria fail to transform daidzein into equol under various experimental conditions *in vitro*. These findings exclude any role of bifidobacteria in the production of equol [39]. Thus, the determination of plasma concentrations of equol especially in subjects demonstrating lesser enhancement of isoflavone bioavailability after FOS supplement should be further studied. However, the presentation of biologically active equol in plasma after FOS supplement, if any, might be able to produce beneficial effects in postmenopausal women despite no significant increase in the bioavailability of daidzein, because equol possesses more estrogenic activity than daidzein and binds more strongly to the estrogen receptors [40]. It also has a longer half-life and superior antioxidant activity [41, 42].

Since the second peak of both isoflavones was demonstrated to be at approximately 2 h after lunch in most subjects enrolled in this study, this finding indicates that the *β*-glucuronide and sulfate conjugates excreted via bile during lunch time might be further deconjugated by the gut microflora and undergo enterohepatic recirculation as suggested by previous studies [43, 44]. This additional absorption of isoflavones into systemic circulation possibly accounts for the second surge of plasma concentrations [29]. Whether stimulation of bacterial growth after FOS administration contributes to enhancement of gut microflora *β*-glucuronidase activity, and hence increase in enterohepatic recirculation during the second peak, is still equivocal and warrants further investigation.

In the present study, continuous administration of FOS could increase AUC of daidzein and genistein by approximately 15% and 30%, respectively, whereas other studies showed that doubling the oral dose of soy nuts from 20 to 40 g yields increments of AUC by approximately 80% and 40%, respectively [30]. Therefore, increased oral ingestion of isoflavones or soy foods seems to be a simple but more effective and less costly means to enhance oral isoflavone bioavailability in comparison to coadministration of fixed-dose isoflavones plus FOS. Nonetheless, FOS has been found to increase intestinal absorption of calcium in rats [45] and humans [46, 47], presumably by stimulating growth of resident microflora such as bifidobacteria. In addition, soy foods combined with a prebiotic significantly improve the lipid profiles in hyperlipidemic adults, possibly via an increase in colonic microbial biotransformation of isoflavones and/or specific short-chain fatty acids [48]. Therefore, coadministration of isoflavones plus FOS might play some advantageous role in certain circumstances, such as in postmenopausal women with coexisting osteoporosis and/or dyslipidemia.

The major limitations of this study were listed as follows. Firstly, since the carry-over effect of FOS on enhancement of bacterial growth especially *in vivo* is not well understood, the study was therefore designed as fixed-sequence rather than a randomized-sequence cross-over study in order to avoid any possible residual effect of FOS. Secondly, a further limitation is the lack of quantification of fecal bacterial counts, *β*-glucosidase activity in the fecal content, and plasma levels of equol.

## 5. Conclusion

Continuous oral administrations of FOS followed by a single oral administration of soy beverage caused significant increases in *C*_max_, AUC_0–32_, and AUC_0–∞_ of genistein and AUC_0–32_ of daidzein, compared to those obtained following a single oral dose of soy beverage alone.

## Figures and Tables

**Table 1:** The demographic characteristics of 12 subjects enrolled in this study.

Subject	Age	Weight	Height	BMI	FSH
No.	(y)	(kg)	(m)	(kg/m^2^)	(mIU/mL)
1	56	57.00	1.52	24.67	41.56
2	58	48.50	1.47	22.44	73.46
3	64	59.00	1.60	23.05	61.71
4	62	55.00	1.51	24.12	60.78
5	50	58.50	1.65	21.49	90.77
6	55	53.00	1.52	22.44	86.68
7	54	59.00	1.54	24.88	79.67
8	55	56.00	1.53	23.92	90.55
9	53	48.50	1.52	20.99	125.84
10	46	54.00	1.55	22.48	30.53
11	67	42.00	1.46	19.70	80.14
12	54	51.00	1.47	23.60	57.87

Mean	56.17	53.46	1.53	22.81	73.30

SD	5.87	5.18	0.05	1.55	25.22

**Table 2:** Pharmacokinetic parameters of daidzein and genistein obtained from 12 subjects receiving a single oral administration of soy beverage (single ISO phase) and continuous oral administrations of FOS following by a single oral administration of soy beverage (continuous FOS/single ISO phase).

Pharmacokinetic parameters	Daidzein		Genistein	
	Single ISO	Continuous FOS/single ISO	Single ISO	Continuous FOS/single ISO
C_max_ (ng/mL)	363.33 ± 116.67	412.90 ± 100.61	347.01 ± 143.42	489.65 ± 187.15*
AUC_0–32_ (ng·h·mL)	2753.71 ± 1227.64	3177.32 ± 1474.55*	4096.66 ± 2050.96	5354.39 ± 2650.16*
AUC_0–∞_ (ng·h·mL)	3399.35 ± 1261.82	3741.85 ± 1421.28	5075.62 ± 2787.77	6321.17 ± 3047.63*
*T*_ max_ (h)	6.67 ± 1.78	6.67 ± 1.78	6.00 + 1.91	6.67 ± 1.56
*t*_1/2_ (h)	4.65 ± 2.46	5.27 ± 2.50	9.61 ± 4.45	10.01 ± 4.17

Data represents mean ± SD. *P < 0.05 versus single ISO phase.
